# Contemporary gene flow between wild *An. gambiae s.s.* and *An. arabiensis*

**DOI:** 10.1186/1756-3305-7-345

**Published:** 2014-07-24

**Authors:** David Weetman, Keith Steen, Emily J Rippon, Henry D Mawejje, Martin J Donnelly, Craig S Wilding

**Affiliations:** Department of Vector Biology, Liverpool School of Tropical Medicine, Pembroke Place, Liverpool, L3 5QA UK; Infectious Diseases Research Collaboration, Makerere University, Kampala, Uganda; Malaria Programme, Wellcome Trust Sanger Institute, Hinxton, Cambridge CB10 1SJ UK; School of Natural Sciences and Psychology, Liverpool John Moores University, Byrom Street, Liverpool, L3 3AF UK

**Keywords:** Introgression, Hybridisation, Backcrossing, Mosquito, Haldane’s rule

## Abstract

**Background:**

In areas where the morphologically indistinguishable malaria mosquitoes *Anopheles gambiae* Giles and *An. arabiensis* Patton are sympatric, hybrids are detected occasionally via species-diagnostic molecular assays. *An. gambiae* and *An. arabiensis* exhibit both pre- and post-reproductive mating barriers, with swarms largely species-specific and male F1 (first-generation) hybrids sterile. Consequently advanced-stage hybrids (back-crosses to parental species), which would represent a route for potentially-adaptive introgression, are expected to be very rare in natural populations. Yet the use of one or two physically linked single-locus diagnostic assays renders them indistinguishable from F1 hybrids and levels of interspecific gene flow are unknown.

**Methods:**

We used data from over 350 polymorphic autosomal SNPs to investigate post F1 gene flow via patterns of genomic admixture between *An. gambiae* and *An. arabiensis* from eastern Uganda. Simulations were used to investigate the statistical power to detect hybrids with different levels of crossing and to identify the hybrid category significantly admixed genotypes could represent.

**Results:**

A range of admixture proportions were detected for 11 field-collected hybrids identified via single-locus species-diagnostic PCRs. Comparison of admixture data with simulations indicated that at least seven of these hybrids were advanced generation crosses, with backcrosses to each species identified. In addition, of 36 individuals typing as *An. gambiae* or *An. arabiensis* that exhibited outlying admixture proportions, ten were identified as significantly mixed backcrosses, and at least four of these were second or third generation crosses.

**Conclusions:**

Our results show that hybrids detected using standard diagnostics will often be hybrid generations beyond F1, and that in our study area around 5% (95% confidence intervals 3%-9%) of apparently ‘pure’ species samples may also be backcrosses. This is likely an underestimate because of rapidly-declining detection power beyond the first two backcross generations. Post-F1 gene flow occurs at a far from inconsequential rate between *An. gambiae* and *An. arabiensis*, and, especially for traits under strong selection, could readily lead to adaptive introgression of genetic variants relevant for vector control.

**Electronic supplementary material:**

The online version of this article (doi:10.1186/1756-3305-7-345) contains supplementary material, which is available to authorized users.

## Background

Across much of sub-Saharan Africa the major malaria vectors are *Anopheles gambiae* Giles, *An. coluzzii* Coetzee & Wilkerson and *An. arabiensis* Patton, members of the morphologically indistinguishable *Anopheles gambiae sensu lato* species complex [1]. Whilst *Anopheles gambiae s.s.* (henceforth *An. gambiae*) is the dominant malaria vector in many areas, there is evidence that in some areas in East Africa [2–5] and urban West Africa [6] *An. arabiensis* is increasing in relative frequency, with a concomitant potential increase in importance for malaria transmission.

The existence of previously unrecognised divisions within the *An. gambiae s.l.* complex were first noted over 50 years ago when crosses between field-collected samples showed that F1 males were sterile and exhibited atrophy of the testes, though F1 females were apparently viable [7]. Since males are the heterogametic sex in *Anopheles* this is in accordance with Haldane’s rule, a well-known form of reproductive isolation observed between recently-diverged species [8, 9]. In addition to first generation hybrid (F1) male sterility, Slotman *et al*. [10] demonstrated that additional inviability effects may occur, due to recessive factors located on the X chromosome of *An. gambiae* which are incompatible with at least one factor on each autosome of *An. arabiensis*. Pre-zygotic isolating mechanisms are also known: under experimental conditions the species mate assortatively [11], which could maintain reproductive isolation when *An. gambiae* and *An. arabiensis* co-occur within the same mating swarms in the wild [12] although the assortative mating cues which limit hybridisation outdoors in the wild can apparently be over-ruled when mosquitoes enter houses [13]. The cues used for species recognition remain unclear. A plausible driver of assortative mating in mixed swarms might be differing wing beat frequencies [14] although direct evidence for this is lacking [15]. The existence of both extrinsic pre-mating barriers and intrinsic post-mating barriers suggests that interspecific gene-flow could be minimal, with few F1 hybrids and a negligible level of further hybridisation by backcrossing to the parental species. Screening of field-collected samples in areas of sympatry does indeed detect hybrids at only low frequencies: 0.02-0.76% [16–18]. However, the standard single-locus diagnostics used, which both target SNPs located near the centromere of the X chromosome [19, 20], and exhibit near-perfect linkage disequilibrium [19], are incapable of discriminating F1s from backcrosses.

Whilst such data argue that advanced backcrossing between *An. gambiae* and *An. arabiensis* should be rare, evidence from both laboratory crosses and inferential data on introgression from field-collected material suggest that this can occur; in inter-specific laboratory mating, whilst there are >80% sterile males in early generations this proportion declines to around 10% after several generations (see Coz, 1973 referred to in [21]) suggesting that if backcrossing of F1s occurs, subsequent hybrid generations might largely overcome sterility barriers. In more recent laboratory interspecific crossing, introgression of shared chromosomal inversions occurred [22] though with different rates of persistence across chromosomes [23] and, in the wild, consensus of evidence suggests that the 2La inversion appears to have introgressed from the aridity-tolerant *An. arabiensis* to *An. gambiae* [24–26] and such introgression requires backcrossing. However, laboratory studies are not necessarily representative of the contemporary situation in wild populations, where interspecific mating will be much rarer, but selection (e.g. through insecticidal pressure) might drive even infrequently introgressed adaptive variation to appreciable frequencies in the recipient species [27].

Genetic analysis of field-collected material has provided indirect evidence consistent with the occurrence of contemporary or recent introgression via patterns of sharing of haplotypes at four nuclear loci [28], in mtDNA [29, 30], in the 2Rb and 2La inversions [31, 32], and from a high ratio (13:1) of shared to fixed polymorphisms located throughout the genome [33]. However, these studies provide only indirect evidence because hybrid individuals were not typed directly and/or the numbers of markers were limited. Accurate determination of the extent of admixture ideally requires large numbers of markers [34] and inclusion of individuals typing as hybrids.

This study takes advantage of a collection of hybrid specimens detected via screening of a very large number (>7,000) of *An. gambiae s.l.* individuals from Eastern Uganda, where hybrids were found at a frequency of 0.22% [18]. We examine multi-locus SNP genotypes of these field-collected hybrids (*N* = 11) of *An. gambiae* and *An. arabiensis* and compare them to PCR-diagnostically pure forms to examine the nature of hybrid detection and interspecific gene flow.

## Methods

### Samples and genotyping

Samples were derived from collections in Uganda; from Jinja in 2011 [18] and Tororo in 2008 and 2009 (Weetman *et al*., unpublished). In total 199 *An. gambiae* (13 from Jinja and 186 from Tororo), 21 *An. arabiensis* (13 from Jinja and 8 from Tororo) and 12 individuals scored as *An. gambiae* x *An. arabiensis* hybrids (11 from Jinja and 1 from Tororo) were studied.

Species were identified using the rDNA [20] and SINE [19] species diagnostic assays. These assays type markers on the X-chromosome separated by *ca*. 1.4 Mb; due to the physical proximity in an area of low recombination the assays almost always yield congruent results [19]. Hybrids exhibited bands for each species at each of these assays when viewed on agarose gels. Some individuals were also genotyped using a diagnostic for the 2La inversion polymorphism [35].

Samples were genotyped using a custom 1536-SNP, Illumina GoldenGate array. We previously designed and utilised v1.0 of this array [36] to preferentially screen insecticide resistance candidate genes with ≈ 20% of the SNPs located in control, intergenic regions or non-candidate regions distributed through the genome. Version two of the array replaced consistently failing SNPs, and provided more balanced genomic coverage (Additional file 1: Figure S1 and Additional file 2: Table S1).

DNA was extracted using the DNEasy extraction kit (Qiagen) and quantified using the PicoGreen quantification kit (Invitrogen) [37]. Individual mosquitoes typically provide insufficient DNA for the GoldenGate assay [37] and therefore whole genome amplification is required. Whole genome amplification of a total of 50 ng of the extracted DNA was performed using the GenomiPhi V2 DNA amplification kit (GE Life Sciences) and quantification repeated using PicoGreen before dilution to 50 ng/μl. Template for the Illumina GoldenGate assay was 5 μl of this whole genome amplified DNA. The assay was run on an Illumina Beadstation GX following the manufacturer’s protocols. To check for possible contamination which would influence hybrid assessment, we sequenced all 12 hybrid samples and 11 *An. gambiae* using primers C1-J-2182 and TL2-N-3014 [38] with conditions as in [39] to amplify 800 bp of the mitochondrial COI gene, and for which no within-sample heterozygosity should be observed. From a total of 64 polymorphic bases, 22 of the 23 samples sequenced showed no heterozygosity, but one hybrid sample was heterozygous at 73% of the sites. Since this sample clearly represented a mixture of DNAs it was removed from the analysis.

### Data analysis

Genotype calls were made with Beadstudio v3.2 (Illumina Inc.) with all calls checked manually. Although predominantly female samples were used (199 female *An. gambiae*, seven *An. arabiensis* from Jinja, eight *An. arabiensis* from Tororo and 11 hybrids), six *An. arabiensis* samples from Jinja (of 13) were males. Since both males and females were studied, X-chromosome SNPs were excluded from the analysis. From a total of 736 reliably scoreable SNPs on the array [36, 40], 462 autosomal SNPs were identified that were polymorphic and exhibited ≤20% missing data in any sample group (each species and hybrids). These 462 SNPs were used for all analyses (Additional file 1: Figure S1). F_ST_ and diversity statistics for each SNP were calculated from genotypes of PCR diagnostically-pure species using GenAlEx 6.5 [41], and the distance among individual multilocus genotypes visualised using principal coordinates analysis (PCoA), also using GenAlEx 6.5 [41], with default settings. Individual multilocus genotypes comprising of SNPs on chromosome 3 and chromosome arm 2R (see Results) served as input for STRUCTURE 2.3.4 [42] and BAPS 6 clustering and genomic admixture analyses [43, 44]. Though normally applied as alternatives, these two methods were used together because STRUCTURE provides estimated admixture proportions for every individual, whereas BAPS only provides admixture proportions if some evidence of mixture is detected (otherwise a zero is returned) but also provides a probability for a hypothesis of no admixture. The admixture algorithm first estimates which multilocus genotypes show evidence of mixture and the proportion of the genome attributed to each source population, followed by simulation of multilocus genotypes from allele frequencies to determine the posterior probability that putatively mixed genotypes could be found in the source population [43, 44]. For STRUCTURE, admixture was estimated from the mean of ten replicates with 10,000 iterations for burn-in and 20,000 for data-collection, with *k* set to two in every run (to capture each species’ samples: STRUCTURE was not applied to determine the optimum number of clusters). In BAPS, multiple runs with *k* set from 2 to 20 were undertaken to obtain optimum clustering solutions. Settings for the admixture analysis were 100 iterations, 200 or 1000 reference individuals for simulations (see below) for observed data, and 20 iterations for the reference individuals. Since ‘pure’ species determined by single-locus diagnostics might actually be mixed genotypes, we computed an outlier analysis for each set of ‘pure’ species data. Using the proportionate mixture estimates from all data from STRUCTURE, we calculated the absolute deviation from the grand median and multiplied by a constant (*b* = 1.4826) representing the normal distribution to yield a median absolute deviation metric (*MAD*). Outliers were considered as data points whose mixture value was more extreme than 3 × *MAD* (in the direction of the alternate species, which represents a conservative threshold [45]. This method has the advantage over those utilising means and standard deviations of being relatively insensitive to the influence of any outliers in the detection process [45]; calculations were performed in Excel. BAPS admixture analysis was then performed using *An. arabiensis* and *An. gambiae*, following exclusion of outliers, as predefined populations and the outliers and hybrids as the test samples.

Simulations of expected mixture proportions for various classes of hybrid were conducted in Hybridlab [46]. Observed genotype data for the ‘pure’ species samples (i.e. excluding outliers) was first used to generate 100 simulated genotypes of each, which served as the data for production of F1, F2, F3 and first to third generation backcrosses. 100 simulated genotypes were produced for each hybrid class for admixture analysis in BAPS with the simulated ‘pure’ species genotypes as predefined reference populations. To evaluate detection power for each hybrid class we calculated the percentage of significantly mixed individuals, mean admixture proportion, and its deviation from the relevant theoretical expectation: 0.5 for F1, F2, F3; 0.25 for first generation backcrosses (bx1), 0.125 for bx2, and 0.0625 for bx3. Admixture proportions of significantly mixed observed genotypes falling with the range of simulated values were considered potentially representative of the hybrid class. Thus genotypes could in some cases be considered a potential member of multiple classes, in which case their precise hybrid class status could not be determined.

## Results

### Dataset properties and refinement

A total of 462 autosomal SNPs could be reliably scored in both species and were polymorphic in at least one (Additional file 1: Figure S1, Additional file 2: Table S1). Whether measured as number of alleles (*Na*) or heterozygosity (*He*), diversity was much lower in *An. arabiensis* (mean ± 95% confidence interval: *Na* = 1.29 ± 0.041; *He* = 0.091 ± 0.015) than *An. gambiae* (mean ± 95% confidence interval: *Na* = 1.96 ± 0.018; *He* = 0.27 ± 0.016), likely reflecting ascertainment bias resulting from use of an array designed from *An. gambiae* polymorphisms. Interspecific differentiation over all loci was calculated as F_ST_ = 0.128 ± 0.014, with only one autosomal locus representing a fixed difference between the species (Additional file 2: Table S1).

**Figure 1:**
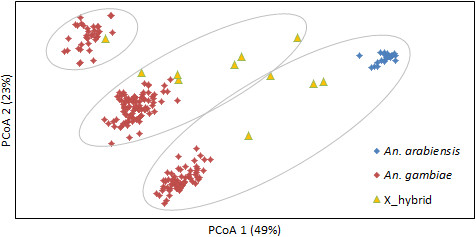
**Principal Coordinates Analysis based on all autosomal SNPs (** ***N*** **= 462).** Ovals showing BAPS clusters based solely on SNPs within the 2La inversion.

Principal coordinates analysis (PCoA) clearly differentiated pure *An. gambiae* from *An. arabiensis*, with hybrids scattered in between (Figure 1). However, hybrid position with respect to the parental species was obscured by subdivision of the *An. gambiae* data points into three groups. Based on previous analyses [33, 36] we hypothesised that this vertical separation results from highly differentiated multilocus genotypes that reflect alternative arrangements of the 21 Mb 2La paracentric inversion on chromosome arm 2L (2La/2La, 2La/2L+^a^, 2L+^a^/2L+^a^). To test this we ran a BAPS clustering analysis using only loci within the 2La inversion (*N* = 61 SNPs). BAPS identified four clusters; two of which overlapped closely but contained a different composition of the species (Figure 1). The three major clusters (counting the overlapping clusters as one) corresponded perfectly with the diagonally-oriented clustering observed using all 462 SNPs, thus these clearly represent the three alternate 2La karyotypic combinations, which we confirmed by genotyping a portion of the individuals (*N*=26; 19 × 2La/2La, 5 × 2La/2L+^a^, 2 × 2L+^a^/2L+^a^) from across the groups using a 2La PCR diagnostic. Owing to this dependence of clustering on 2La genotypes we proceeded with subsequent analysis using only SNP data from chromosome arm 2R and both arms of chromosome 3. PCoA analysis of this reduced dataset (*N* = 353 polymorphic SNPs) demonstrated that the 2La genotype stratification was no longer evident (Additional file 1: Figure S2) and also that samples from the different collection locales were well mixed in clusters (Additional file 1: Figure S3), suggesting this would exert negligible impact on any subsequent analysis.

### Genomic admixture and hybrid classification

**Figure 2:**
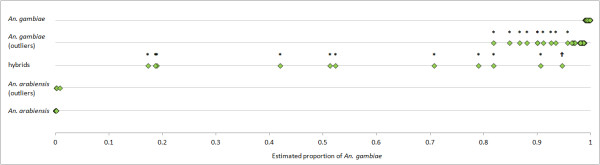
**Proportion of** ***An. gambiae*** **genome estimated in multilocus genotypes (** ***N*** **= 353 SNPs) estimated by STRUCTURE.** Samples are categorised as: ‘pure’ *An. gambiae* or *An. arabiensis* from IGS and SINE diagnostics, and non-outlier status in terms of their proportions of the appropriate genome (top and bottom rows); outliers from the pure species (second and fourth rows) or hybrids (solely) from IGS and SINE diagnostics (middle row). *statistically significant genome mixture detected by BAPS; †marginally non-significant genome mixture. Note that although BAPS and STRUCTURE admixture coefficients were strongly correlated for significantly mixed individuals (Pearson’s r = 0.998, *N* = 22) the relationship between likelihood of significance and the estimated average level of genomic admixture is not expected to be perfect.

Analysis of individual admixture coefficients (proportion of each 353-SNP genotype attributed as of *An. arabiensis* or *An. gambiae* in origin) estimated using STRUCTURE identified two putative *An. arabiensis* and 34 putative *An. gambiae* as outliers (Additional file 2: Table S2). In order to obtain ‘pure’ species data the outliers were excluded. Significance of genomic admixture was assessed using BAPS with ‘pure’ species genotypes (i.e. excluding outliers) as two reference datasets against which patterns of admixture were evaluated in both the outlier samples and the hybrids (pre-identified using X-linked PCR diagnostics). Both *An. arabiensis* outliers and 24 out of 34 of the *An. gambiae* outliers were not adjudged significantly mixed, but 10 out of 11 hybrids were significant, and displayed admixture proportions overlapping those of the ten significantly mixed *An. gambiae* outliers. Thus, hybrids formed a spectrum of admixture, and only a minority (three of 11) were close to the 50:50 that would be expected for F1 hybrids (Figure 2).

**Figure 3:**
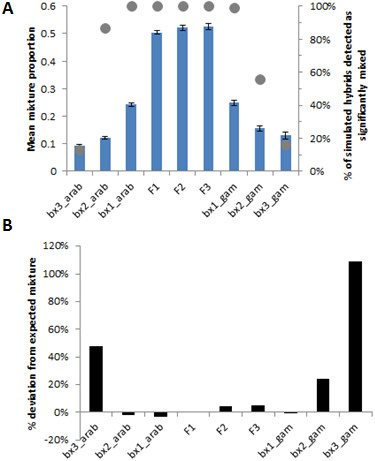
**Hybridlab results for simulation of different levels of intercrossing and backcrossing to each parental species.** Simulations were undertaken for different levels of intercrossing (F) and backcrossing (bx) **(A)** Dots show the % of hybrids exhibiting significant genomic admixture; bars show mean genomic admixture proportions for simulated individuals detected as significantly admixed (+/- 95% confidence interval). **(B)** Bias in mean admixture proportion for individuals detected as significantly mixed, expressed as deviation of observed data from the theoretical expectation for the level of crossing (i.e. F1 = 0.5; first generation backcross = 0.75, etc.).

In order to evaluate the power of hybrid detection and to produce empirical categories to which observed data could be fitted, simulations were run in HybridLab. The expected proportions of the genome originating from each parental species and probability of detection of significant admixture were computed using BAPS for nine different cross scenarios (Figure 3). As expected, F1 hybrids are indistinguishable from advanced intercrosses (F2, F3), but power to detect intercross classes and also first generation backcrosses was 99-100% (Figure 3A) with minimal deviation of simulated admixture proportions from theoretical expectations (Figure 3B). Power to detect second generation backcrosses was much higher for crosses with *An. arabiensis* than *An. gambiae*, but was very low (<20%) for the third generation (Figure 3A) and detection strongly biased toward those exhibiting relatively greater admixture (Figure 3B).

Plausible hybrid ancestries for the outliers and pre-identified hybrids are shown in Table 1 and range through all potential scenarios from F1 to advanced back-crosses, with all ten outliers identifiable as backcrosses to *An. gambiae.* Overall, only one third of the pre-diagnosed hybrid samples appear to represent F1 hybrids (Table 2), with confidence intervals suggesting between approximately 31-89% are likely to be backcrosses, with up to 9% of *An. gambiae* diagnosed as pure species by X-diagnostics (Table 2). The marginally non-significant hybrid sample most likely represents a type II error arising from poor detection power for advanced backcross genotypes with such a concomitantly low level of mixture.

**Table 1:** **Classification of hybrid category for individuals detected as significantly mixed in Hybridlab/BAPS analyses**

				bx3_*A.a*	bx2_*A.a*	bx1_*A.a*	F1	F2	F3	bx1_*A.g*	bx2_*A.g*	bx3_*A.g*	
			Sim_max	0.92	0.92	0.84	0.58	0.64	0.64	0.36	0.26	0.18	
			Sim_min	0.89	0.81	0.66	0.41	0.41	0.41	0.16	0.09	0.09	
IGS & SINE	Arab	Gam	Prob (pure)										
*A.g*	0.16	0.84	<0.001	0	0	0	0	0	0	1	1	1	bx1+
*A.g*	0.12	0.88	0.006	0	0	0	0	0	0	0	1	1	bx2+
*A.g*	0.18	0.82	<0.001	0	0	0	0	0	0	1	1	1	bx1+
*A.g*	0.16	0.84	<0.001	0	0	0	0	0	0	1	1	1	bx1+
*A.g*	0.15	0.85	<0.001	0	0	0	0	0	0	0	1	1	bx2+
*A.g*	0.19	0.81	<0.001	0	0	0	0	0	0	1	1	0	bx1or2
*A.g*	0.23	0.77	<0.001	0	0	0	0	0	0	1	1	0	bx1or2
*A.g*	0.14	0.86	0.001	0	0	0	0	0	0	0	1	1	bx2+
*A.g*	0.14	0.86	0.001	0	0	0	0	0	0	0	1	1	bx2+
*A.g*	0.16	0.84	<0.001	0	0	0	0	0	0	1	1	1	bx1+
Hybrid	0.81	0.19	<0.001	0	1	1	0	0	0	0	0	0	bx1or2
Hybrid	0.50	0.50	<0.001	0	0	0	1	1	1	0	0	0	F1+
Hybrid	0.23	0.77	<0.001	0	0	0	0	0	0	1	1	0	bx1or2
Hybrid	0.53	0.47	<0.001	0	0	0	1	1	1	0	0	0	F1+
Hybrid	0.15	0.85	0.004	0	0	0	0	0	0	0	1	1	bx2+
Hybrid	0.82	0.18	<0.001	0	1	1	0	0	0	0	0	0	bx1or2
Hybrid	0.07	0.93	0.058†	0	0	0	0	0	0	0	0	0	see text
Hybrid	0.22	0.78	<0.001	0	0	0	0	0	0	1	1	0	bx1or2
Hybrid	0.31	0.69	<0.001	0	0	0	0	0	0	1	0	0	bx1
Hybrid	0.81	0.19	<0.001	0	1	1	0	0	0	0	0	0	bx1or2
Hybrid	0.58	0.42	<0.001	0	0	0	1	1	1	0	0	0	F1+

Header rows show the maximum and minimum proportions for each category of hybrid simulated, where e.g. bx3_*A.a* is a third backcross generation to *An. arabiensis.* X-diagnostic marker classification of each sample is shown in the far left column followed by the BAPS estimate of the proportion of each species present in the sample and associated probability that the sample is not mixed. A ‘1’ under any category indicates the sample could represent a member of the hybrid category based on simulations. The far right column is a summary of plausible hybrid categories for each sample, where e.g. bx1+ is a backcross of the first or subsequent generation.

**Table 2:** **Hybridlab/BAPS classification of samples characterised as hybrids or pure species using X-diagnostic markers**

IGS & SINE	F1+	bx_*A.g*	bx_*A.a*	NS	p(bx)	LCL95	UCL95
hybrid	3	4	3	1^†^	0.700	0.354	0.919
*A.a*				21	0	0	0.161
*A.g*		10		189	0.050	0.024	0.091

Samples were characterised using two X-chromosome diagnostic markers – the IGS [20] and SINE [19]. bx_*A.a* and bx_*A.g* are are backrosses to *An. arabiensis* and *An. gambiae*. Counts in columns 2–4 show significantly mixed individuals; NS = not significantly mixed. p(bx) and LCL95, UCL95 are the frequency of backcrosses and associated binomial lower and upper confidence limits. ^†^ *P* = 0.058; note > bx3_*A.g* are below detection limit of test.

## Discussion

Even in the absence of intrinsic post-zygotic isolating mechanisms, selection against F1 genotypes can be strong if parental species display ecological niche segregation [47], which is at least partially true of *An. gambiae s.s.* and *An. arabiensis* [48, 49]. When coupled with male sterility, as predicted by Haldane’s rule [9], it would seem entirely possible that hybrids between these *An. gambiae s.l.* species might be near-ubiquitously F1s and a near dead-end for contemporary gene flow. Our results show that this is definitely not the case. Although hybrids are rare, where detected there is a 30-90% probability (confidence intervals from our data) that they will be backcrosses, some quite advanced. These results highlight that an important conduit for gene flow exists between species which could permit adaptive introgression of genetic variants [50].

Convincing demonstrations of contemporary adaptive introgression between animal species are very rare [50] though transfer of anticoagulant rodenticide resistance from *Mus spretus* to *Mus musculus* [27] - which also exhibit Haldane’s rule and are subject to strong anthropogenic selection pressure - provides a comparable, if phylogenetically-disparate, case study. Indeed transfer of the strongly-selected *Vgsc-1014F* mutation from *An. gambiae s.s.* (S form) to *An. coluzzii* (M form), with a subsequent dramatic increase in frequency [51], has been unambiguously demonstrated [36, 52]. *An. gambiae s.s.* and *An. coluzzii* exhibit similar partial ecological niche separation and hybrid and backcross detection rates appear broadly comparable to those found in the present study [40, 53]. Does this similarity, coupled with the results presented here mean that adaptive introgression between *An. gambiae* and *An. arabiensis* will occur, or perhaps has already done so, in response to anthropogenic selection? The *An. arabiensis* samples from Jinja studied here were from insecticide resistance-phenotyped specimens [18], but we have yet to identify the mechanisms involved. Kawada *et al*. [54] report introgression of *Vgsc-1014S* from *An. gambiae* to *An. arabiensis* in samples from neighbouring Kenya. Yet their sequencing of the intron downstream of *Vgsc-1014S* detected insufficient variation to support this conclusion, and Kawada *et al*.’*s* [54] study actually provides no evidence for introgression. The *Vgsc-1014F* mutation has been identified in West African *An. arabiensis* but this is a *de novo* phenomenon and not introgression from *An. gambiae* [55].

Adaptive introgression is likely to involve massive disruption of the recipient genome, because selection will tend to cause a sweep of an extended region of the source genome through the population as observed in both *Mus domesticus* and *An. coluzzii* [27, 51]. Such introgressed genomic regions may contain many variants that are maladaptive for the recipient genome and recombination will take time to reduce the region size to retain only the beneficial locus [50]. Though apparently selectively advantageous for *An. coluzzii*, this species is much more closely related to *An. gambiae s.s.* than is *An. arabiensis* [33], and thus potential for disruption of the genome may be more limited. At present we know little of the selective advantage or disadvantage experienced by the backcrosses or F1 hybrids we detected, which will require identification of a selected introgressed locus, or direct experimental testing of relative fitness. Nevertheless our results have established that the potential exists for adaptive introgression; whether this occurs will depend on the balance of the positive selective coefficient of the adaptive locus (or loci), the (assumed) negative selective coefficient of the other variants in the introgressed fragment, and the background recombination rate of the introgressed region [53].

This study was enabled by genotyping of very large numbers of each species at the single locus species-diagnostic markers to identify hybrids, and subsequent genotyping at a relatively large number of genomewide SNP markers, and directly demonstrates post-F1 gene flow between *An. arabiensis* and *An. gambiae* in the wild. Our results are consistent with indirect evidence of introgression from previous molecular genetic and cytogenetic studies [28–33] and with direct evidence of introgression from laboratory mating [23]. In spite of a relatively large number of markers genotyped, power to detect backcrossing became severely limited by the third generation and future studies of introgression will benefit from availability of whole genome sequence datasets for each species, as well as providing estimates of differentiation throughout the genome that are unaffected by the ascertainment bias observed here and evident in a previous genomewide SNP study [56]. Moreover, genotyping by sequencing should permit identification of many markers exhibiting fixed differences, which can provide a diagnostic panel to study backcrossing [57]. Here we identified only one fixed difference, which can provide little additional discrimination, because six or more fully diagnostic markers are required to statistically partition hybrids as F1s and backcrosses (based on binomial probabilities and a threshold *P* of 0.05).

## Conclusion

Our study demonstrates unambiguously the occurrence of introgressive hybridisation between *An. gambiae* and *An. arabiensis*. To fully understand the adaptive and applied importance of this observation additional studies are required, preferably involving whole genome sequencing. The *An. gambiae* genome has been sequenced [58] and the *An. arabiensis* genome is now available [59]. These data will aid in understanding the extent of genomic differentiation and, as additional *An. gambiae* and *An. arabiensis* whole genome sequences are made available (e.g. [60]), the extent of genomic introgression will be revealed in detail.

## Electronic supplementary material

Additional file 1: Figure S1: Graphical representation of SNPs scored on the array and those used in the study analysis (following exclusion of monomorphic SNPs and those with higher rates of missing values). Each cross is a SNP with position representing physical position in the genome. Grey lines are divisions between chromosomes. See Additional file 2: Table S1 for a full list of SNPs. **Figure S2.** PCoA of autosomal SNPs (N=353) excluding those from chromosome arm 2L. The key is the same as Figure 1 (red = *An. gambiae*; blue = *An. arabiensis*; yellow = X_hybrids). **Figure S3.** PCoA of autosomal SNPs excluding those from chromosome arm 2L with samples split by sample site/time. (DOCX 94 KB)

Additional file 2: Table S1: SNPs used in the study and associated polymorphism and differentiation statistics. **Table S2.** Estimated proportion of *An. gambiae* genome in 353-SNP genotypes using STRUCTURE (mean and stdev of 10 runs). Outliers were classified as those samples >3 absolute deviations from the grand median (calculated separately for each species). Note that hybrids were not included in either median calculation but were classed as outliers based on deviation from either species (*An. gambiae* median deviations shown). (XLSX 59 KB)

## Abbreviations

BAPS: Bayesian analysis of genetic population structure
*Kdr*: Knockdown resistance
Mb: Mega base pairs (=1,000,000 bp)
mtDNA: Mitochondrial DNA
PCoA: Principal coordinates analysis
PCR: Polymerase chain reaction
rDNA: Ribosomal DNA
SINE: Short interspersed nuclear element
SNP: Single nucleotide polymorphism.
