# Supplementary figures and images for: Contemporary gene flow between wild An. gambiae s.s. and An. arabiensis
Source: Parasit Vectors. 2014 Jul 24;7:345. doi: 10.1186/1756-3305-7-345 (PMC4124135; doi:10.1186/1756-3305-7-345)

Supplementary Figure 1.

Supplementary Figure 2


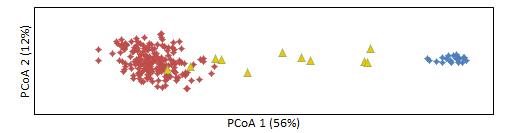


Supplementary Figure 3


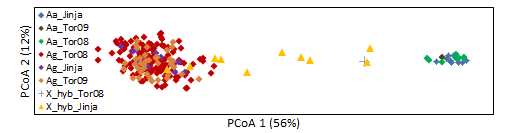

Supplement: Supplementary file 1 — Additional file 1: Figure S1: Graphical representation of SNPs scored on the array and those used in the study analysis (following exclusion of monomorphic SNPs and those with higher rates of missing values). Each cross is a SNP with position representing physical position in the genome. Grey lines are divisions between chromosomes. See Additional file 2: Table S1 for a full list of SNPs. Figure S2. PCoA of autosomal SNPs (N=353) excluding those from chromosome arm 2L. The key is the same as Figure 1 (red = An. gambiae; blue = An. arabiensis; yellow = X_hybrids). Figure S3. PCoA of autosomal SNPs excluding those from chromosome arm 2L with samples split by sample site/time. (DOCX 94 KB) [file 13071_2014_1535_MOESM1_ESM.docx]
